# Bidirectional crosstalk between cancer cells and cancer‐associated fibroblasts in mixed organoid system elicits transcriptomic characteristics of pancreatic cancer with potential therapeutic vulnerabilities

**DOI:** 10.1002/ctm2.1597

**Published:** 2024-02-22

**Authors:** Jae‐Il Choi, Min Jae Yang, Kyoung‐Jin Woo, So Hyun Park, Dakeun Lee, Seokhwi Kim

**Affiliations:** ^1^ Department of Pathology Ajou University School of Medicine Suwon South Korea; ^2^ Department of Biomedical Sciences Ajou University Graduate School of Medicine Suwon South Korea; ^3^ Department of Internal Medicine Ajou University School of Medicine Suwon South Korea

Dear Editor

Recent advancements in organoid technology enable tailored medicine for clinically devastating pancreatic cancer.^1^, ^2^, ^3^ The correlation between the responsiveness of patient‐derived pancreatic cancer organoids (PCOs) to chemotherapy suggests their potential as predictive instruments for therapeutic responses.^4^, ^5^ However, a PCO, solely composed of pancreatic cancer cells and lacking the interplay within the tumour microenvironment (TME), comes with inherent limitations: first, the understanding of non‐genomic characteristics acquired through cellular interplay remains unclear, and secondly, it may exhibit abundant organoid‐specific genes, resulting in a skewed transcriptomic and phenotypic disposition disparate from the patient profile.^6^ Cancer‐associated fibroblasts (CAFs) play a pivotal role in pancreatic cancer. A composite organoid model encompassing both cancer cells and CAFs has unveiled a propensity for chemoresistance to conventional treatments,^7^, ^8^ which is absent within cancer‐only organoids.^9^ However, the intricate interplay between cancer cells and CAFs within this mixed PCO‐CAFs remains to be explored.

This study introduces a comprehensive transcriptomic analysis of the mixed PCO‐CAFs, comparing them with both PCOs and patient tumour tissues. We also identify potential therapeutic targets through this analysis, to provide new avenues for therapeutic intervention.

We generated five corresponding sets of patient tumor tissue, patient‐derived PCO, and mixed PCO‐CAF as previously outlined (Figure 1A, Table S1).^10^ The mixed PCO‐CAF consisted of pancreatic cancer cells and CAFs (Figure S1A), closely resembling histologic features observed in the corresponding patient tissue specimens (Figure 1B). Through bulk RNA sequencing analysis, we substantiated that the mixed PCO‐CAF exhibits a greater resemblance to the transcriptomic characteristics inherent to those patient tissues compared to PCOs when analyzed with the top 500 variable genes identified in each sample (Figures 1C, S2 and Table S2).

**FIGURE 1:**
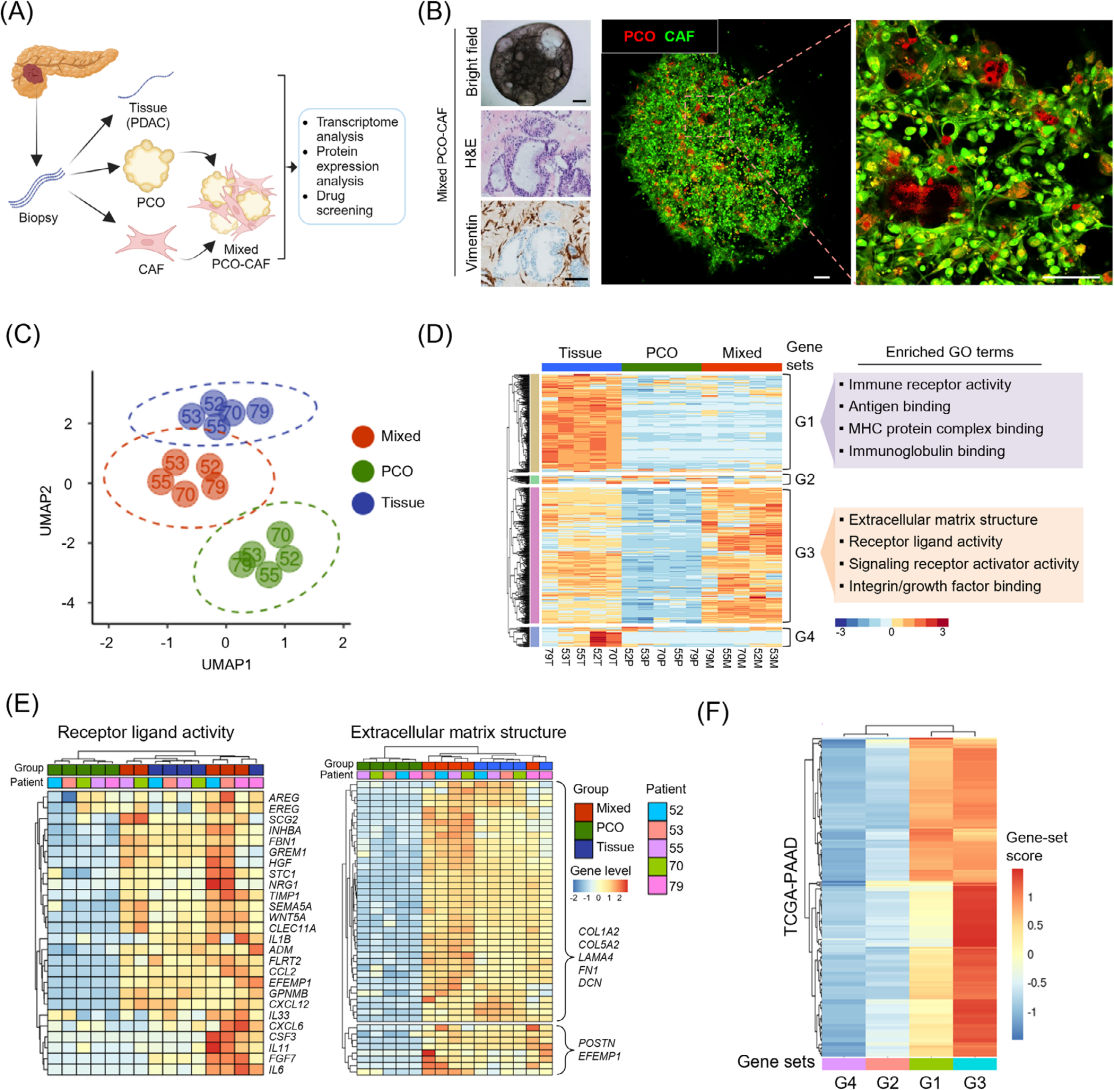
The transcriptomic characterization of the mixed pancreatic cancer organoid‐cancer‐associated fibroblast system. (A) Schematic diagram of establishment and analyses of mixed pancreatic cancer organoid (PCO)‐cancer‐associated fibroblast (CAF). (B) Representative bright field (left upper, scale bar, 200 μm), H&E staining (left mid), vimentin immunohistochemical staining (left lower, scale bar, 500 μm), and immunofluorescence image (right, scale bar, 100 μm) of the mixed PCO‐CAF. PCOs are labelled with deep red and CAFs are labelled with CFSE. (C) UMAP analysis using the top 500 variable genes in the patient tissues, mixed PCO‐CAFs, and PCOs. The ellipses indicate a multivariate normal distribution for the group. The number indicates the patient number. (D) Heatmap showing the expression level divided into four clusters for the top 500 variable genes, with enriched GO terms in G1 and G3. (E) Heatmap showing the expression level of genes in “Receptor ligand activity” (left) and “Extracellular matrix structure” (right) GO across the five pairs of the patient tissue, mixed PCO‐CAF, and PCO. (F) RNA sequencing result of TCGA‐PAAD (*n* = 178) revealing the enrichment of G1 and G3 cluster. CAF, cancer‐associated fibroblast; H&E, hematoxylin and eosin; PCO, pancreatic cancer organoid; PDAC, pancreatic ductal adenocarcinoma; UMAP, Uniform Manifold Approximation and Projection.

Among the four clustered gene sets based on their expression levels (Figure 1D), Geneset 1 (G1) comprised 187 genes that displayed up‐regulated immune response‐related pathway gene ontology exclusively within the patient tissue (Figure S3A). Conversely, Geneset 3 (G3), which comprised 260 genes, exhibited comparable up‐regulation both in the patient tissue and the mixed PCO‐CAF compared to the PCO. Within G3, the elevated expression of diverse growth factors and cytokines encompassed within the Receptor‐ligand activity and the extracellular matrix (ECM) structure gene ontology, highlighting their integral involvement with cellular communication and matrix‐related activities, were consistently discerned within both the patient tissue and the mixed PCO‐CAFs (Figure 1E and S3B). These findings suggest that the mixed PCO‐CAF not only facilitates the emulation of a stroma‐rich TME through ECM production but also orchestrates intercellular signalling communication by virtue of the expression of ligands and corresponding receptors. Furthermore, analysis of RNA sequencing data sourced from The Cancer Genome Atlas (TCGA) dataset for pancreatic adenocarcinoma corroborates the significant enrichment of G3 (Figure 1F).

A single cell‐level transcriptome analysis was conducted on the PCO, mixed PCO‐CAF, and the corresponding patient tissue. The cellular composition within the mixed PCO‐CAF encompassed cancer cells and CAFs of distinct subtypes, comprising myofibroblast‐like CAFs (myCAFs; CAF‐1 cluster) and inflammatory CAFs (iCAFs; CAF‐2 and CAF‐3 clusters) (Figure 2A,B). To validate the similarity between cancer cells from tissue and those from PCO‐CAF, we integrated cancer cell subsets from the matched patient sample. The Uniform Manifold Approximation and Projection and trajectory analysis unveiled discernible clustering of cancer cells within the mixed PCO‐CAF, distinguishing them from those within the PCO, and that the cancer cells from the mixed PCO‐CAF exhibited a transition towards states closely resembling those from the patient's tissue (Figure 2C,D). While the cancer cells present in both the mixed PCO‐CAF and the patient tissue displayed an enrichment of genes associated with growth factor activity and ECM interactions, the cancer cells within the PCO displayed heightened expression of genes linked to cell division mechanisms (Figure 2E,F and Figure S4A–D). Significant enrichment of gene signatures such as TNFα signalling via NF‐κB and epithelial‐mesenchymal transition (EMT) was discerned within the cancer cells of the mixed PCO‐CAF (Figure 2G,H). Especially, cancer cells in the mixed PCO‐CAF showed increased expression of EMT‐related genes, such as MMP1 and vimentin, while the expression of epithelial markers was decreased (Figure 2I). Further comparison between the cancer cells in the mixed PCO‐CAF and the PCO mono‐culture revealed that these alterations are partly derived from the secreted factors in the culture medium (Figure 2J,K and Figure S4E). These findings collectively propose a dynamic reshaping of the transcriptomic landscape within cancer cells in the mixed PCO‐CAF system, attributable to the bidirectional communication established between cancer cells and CAFs.

**FIGURE 2:**
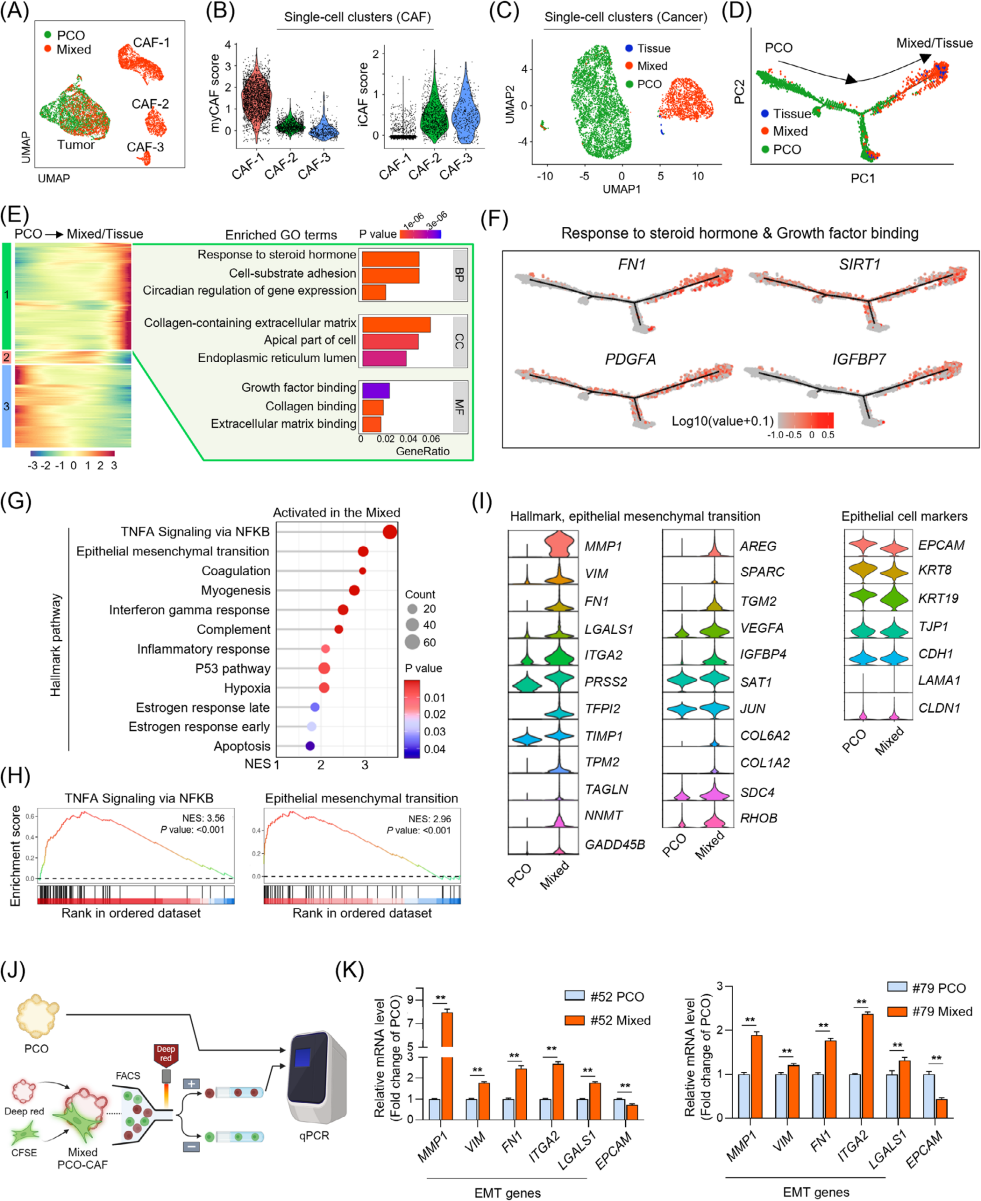
The transcriptomic reshaping within cancer cells through intercellular crosstalk in the mixed pancreatic cancer organoid‐cancer‐associated fibroblast system. (A) UMAP plot of pancreatic cancer organoid (PCO) (green) and mixed PCO‐cancer‐associated fibroblast (CAF) (red) from the single‐cell RNA sequencing data of patient #52. (B) Violin plots showing the enrichment of myCAF (left) and iCAF score (right) in the three CAF clusters (CAF‐1, 2 and 3). (C) UMAP plot of cancer cells in the patient tissue (blue), the mixed PCO‐CAF (red), and the PCO (green). (D) Trajectory analysis showing the enrichment of organoid‐specific genes (left region) and altered genes in cancer cells through crosstalk with CAFs (right region). (E) Pseudotime heatmap of differentially expressed 2000 genes, divided into three clusters (left) with enriched GO terms in the cluster enriched in the mixed PCO‐CAF and patient tissue (right). BP, biological process; CC, cellular component; MF, molecular function. (F) Trajectory maps of the representative genes in the ontology of “Response to steroid hormones” (GO:0048545) and “Growth factor binding” (GO:0019838), enriched in the mixed pancreatic cancer organoid and cancer‐associated fibroblast system. (G) Activated hallmark gene sets in pancreatic cancer cells in the mixed PCO‐CAF culture compared to the PCO mono‐culture by gene set enrichment analysis. (H) Gene set enrichment analysis of TNFA Signaling via NFKB gene set and epithelial‐mesenchymal transition gene set in the mixed PCO‐CAF culture compared to the PCO mono‐culture. (I) Elevation of the individual genes of hallmark epithelial‐mesenchymal transition gene set, along with epithelial cell markers in the mixed PCO‐CAF culture and the PCO monoculture. (J) Schematic diagram of the validation of the cancer cell‐specific gene expression analysis in the mixed PCO‐CAF culture and the PCO monoculture. (K) Elevation of epithelial‐to‐mesenchymal transition‐related gene expression and decrease of EPCAM expression in the cancer cells of the mixed PCO‐CAF cultures and those of the PCO mono‐cultures from patient #52 and patient #79. EMT, epithelial‐to‐mesenchymal transition; GO, gene ontology; iCAF, inflammatory CAF; myCAF, myofibroblastic CAF; UMAP, Uniform Manifold Approximation and Projection.

We next focused on identifying cellular interactions within the mixed PCO‐CAF. Notably, the pancreatic cancer cells not only displayed cancer cell‐to‐cancer cell interactions but also a heightened up‐regulation of interaction weights, primarily evident in their interactions with iCAFs in comparison to myCAFs. These interactions were mediated through a multitude of ligand‐receptor pathways, including MIF‐CD74, GRN‐SORT1, GDF15‐TGFR2, HGF‐MET, INHBA‐ACVR, TNC‐SDC and BDNF‐NTRK2, by enhanced expression of ligands within CAFs and the corresponding receptors within the cancer cells (Figure 3A). Several of these ligand‐receptor interactions were identified within the five patient samples and mixed PCO‐CAFs (Figure 3B). When evaluating the therapeutic potential of targeting the aforementioned up‐regulated pathways, cancer cells within the mixed PCO‐CAF exhibited an elevated vulnerability to targeted therapeutics, surpassing the susceptibility observed in the PCO (Figure 3C). The mixed PCO‐CAFs from patient #52 revealed an enhanced response to Trametinib (NRG inhibitor) and Sapitinib (ERBB3 inhibitor) compared to the PCO mono‐culture (Figure 3D,E), as shown by the highlighted NRG‐ERBB3 interactions in the sample (Figure 3A,B). Additionally, the mixed PCO‐CAFs from patients #79 and #70 showed increased susceptibility to Trametinib and/or ANA‐12 (NT inhibitor) (Figure 3F,G and Figure S5), which could be inferred from the sequencing data (Figure 3B). It is noteworthy that the cancer cells within the mixed PCO‐CAF also exhibited heightened resistance to the gemcitabine and paclitaxel, unlike those within the PCO. This resistance could be attributed to the intricate interplay between cancer cells and CAFs, as previously underscored.^7^, ^8^

**FIGURE 3:**
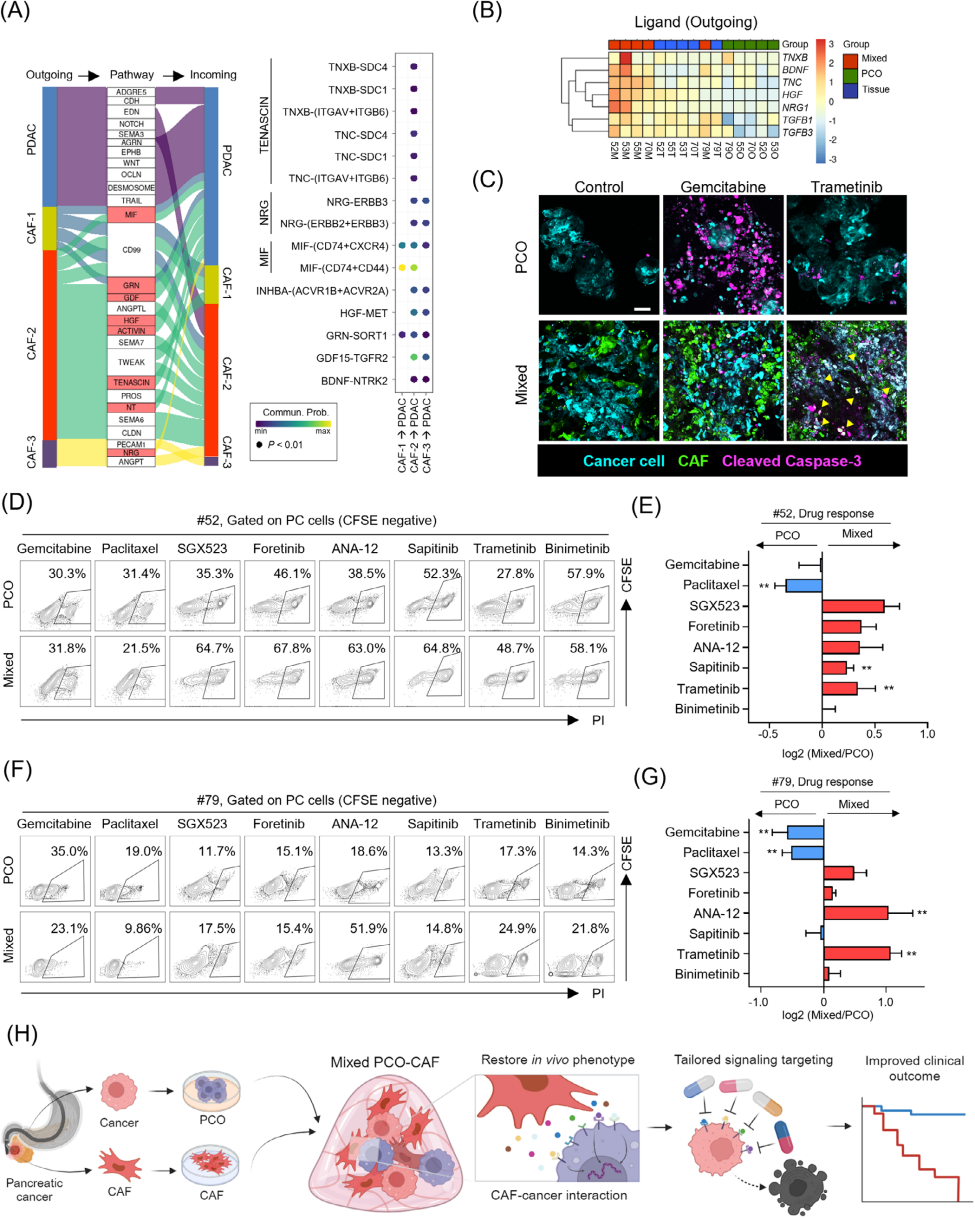
The identification of therapeutic targets by the evaluation of the crosstalk in the mixed pancreatic cancer organoid‐cancer‐associated fibroblast system. (A) Sender‐receiver cell pairs and interacting pathways in the mixed pancreatic cancer organoid (PCO)‐cancer‐associated fibroblast (CAF) (left) and dot plot showing CAF‐to‐cancer cell communication through ligand‐receptor pairs of interacting pathways (right). (B) Heatmap of the up‐regulated ligand (outgoing) genes in the five pairs of the patient tissue (Tissue), the pancreatic cancer‐only organoid (PCO), and the mixed PCO‐CAF (Mixed), which were revealed by the single‐cell RNA sequencing of the sample from patient #52. (C) Representative immunofluorescence images showing drug susceptibility in the PCO and the mixed PCO‐CAF from patient #79. Yellow arrowheads, cancer cells expressing cleaved caspase‐3, implying cell death. (D) FACS plot showing the targeted drug response in the mixed PCO‐CAF and the PCO from patient #52. (E) Quantification of drug response revealing the resistance to conventional drugs and the increased susceptibility to targeted therapeutics in the mixed PCO‐CAF compared to the PCO from patient #52. Data are presented as mean ± SEMs. Significant differences are calculated by t‐test (**, *P* < 0.01). (F) FACS plot showing the targeted drug response in the mixed PCO‐CAF and the PCO from patient #79. (G) Quantification of drug response revealing the resistance to conventional drugs and the increased susceptibility to targeted therapeutics in the mixed PCO‐CAF compared to the PCO from patient #79. Data are presented as mean ± SEMs. Significant differences are calculated by t‐test (**, *p* < 0.01). (H) Schematic diagram of the summary of this study. GO, gene ontology; iCAF, inflammatory CAF; myCAF, myofibroblastic CAF.

In conclusion, our study demonstrated that the mixed PCO‐CAF proficiently recapitulates patient tissue transcriptomic and receptor‐ligand engagements when contrasted with the PCO, offering insights into the intricate intercellular communication between cancer cells and CAFs and personalized therapeutic application (Figure 3I).

## AUTHOR CONTRIBUTIONS

Conceptualization: J‐IC, MJY, DL and SK. Methodology: J‐IC, MJY, K‐JW, SHPand SK. Investigation: J‐IC, KJW, SHP and SK. Formal analysis: J‐IC, MJY, DL and SK. Funding acquisition: MJY, DL and SK. Writing—original draft: J‐IC, DL and SK. Writing—review and editing: J‐IC, MJY, DL and SK. Approval of the final manuscript: all authors.

## CONFLICT OF INTEREST STATEMENT

The authors declare that no conflict of interest.

## ETHICS STATEMENT

This study was approved by the institutional review board of Ajou University Hospital (AJIRB‐BMR‐20‐222). Written informed consent was obtained from all patients. All procedures were in accordance with the Declaration of Helsinki.

## Supporting information

Supporting Information

Supporting Information

FiguresS1‐S5

## Data Availability

RNA‐sequencing data files are deposited in the GEO database (GSE253561). All other data generated in this study are available from the corresponding author upon reasonable request.
