# Supplementary material for: Bidirectional crosstalk between cancer cells and cancer‐associated fibroblasts in mixed organoid system elicits transcriptomic characteristics of pancreatic cancer with potential therapeutic vulnerabilities
Source: Clin Transl Med. 2024 Feb 22;14(2):e1597. doi: 10.1002/ctm2.1597 (PMC10883236; doi:10.1002/ctm2.1597)
Supplement: Supplementary file 1 — Supporting Information [file CTM2-14-e1597-s003.docx]

**Supplementary methods**

**Human material for organoid culture**

Five patients (patient #52, #53, #55, #70, and #79) who were diagnosed with pancreatic adenocarcinoma were selected from our previous study (NCT05571956) (Table S1).^1^ The cases were confirmed to harbor KRAS codon 12 mutation from the formalin-fixed paraffin-embedded blocks by the PNA Clamp KRAS Mutation Detection kit (PANAGENE, Daejeon, Korea) utilizing the real-time polymerase chain reaction. For all patients, two more passages of endoscopic ultrasound-guided fine-needle biopsy (EUS-FNB) were performed beside the sample for histopathologic diagnosis; one for organoid culture and another for RNA sequencing. An additional passage of EUS-FNB was conducted to acquire the fresh sample for single-cell RNA sequencing in patient #52. Written informed consent were obtained from all patients and the study protocol was approved by the institutional review board of Ajou University Hospital (AJIRB-BMR-20-222). This study is registered at <https://clinicaltrials.gov> as NCT05571956.

**Isolation and culture of pancreatic cancer organoid and cancer-associated fibroblast from human sample**

Both pancreatic cancer organoids (PCOs) and cancer-associated fibroblasts (CAFs) were isolated from a single passage of the EUS-FNA specimen. Isolation and culture of PCO and CAFs were conducted by the previously described methods.^1,2^ In brief, about 80% volume of the obtained single-passage FNB sample was utilized for PCO culture. We utilized the culture medium consisting of AdDMEM/F12 medium supplemented with GlutaMAX (Thermo Fisher Scientific, Waltham, MA, USA), penicillin/streptomycin (Thermo Fisher Scientific), B27 (Thermo Fisher Scientific), N-acetyl-L-cysteine (1 mM; Sigma-Aldrich, Saint Louis, MO, USA), Wnt3a-conditioned medium (50% v/v), RSPO1-conditioned medium (10% v/v; R&D Systems, Minneapolis, MN, USA), recombinant noggin protein (100 ng/ml; PeproTech, Rocky Hill, NJ, USA), recombinant epidermal growth factor protein (EGF, 50 ng/mL; PeproTech), gastrin (10 nM; Sigma-Aldrich), recombinant fibroblast growth factor 10 protein (FGF10, 100 ng/mL; PeproTech), nicotinamide (10 mM; Sigma-Aldrich), and A83-01 (0.5 μM; Tocris, Minneapolis, MN, USA) for the PCO culture. The successful culture of more than five passages was regarded as an established PCO. For the isolation and culture of CAF, about 20% volume of the single-passage FNB sample was dissociated using collagenase (Sigma-Aldrich), and the obtained CAFs were cultured in the DMEM/F12 (Thermo Fisher Scientific) with 10% FBS.

**Generation of mixed pancreatic cancer organoid and cancer-associated fibroblast system**

The PCOs were dissociated using TrypLE™ Express (Thermo Fisher Scientific), and CAFs were mixed at a 1:4 ratio. The mixture was cultured on round-bottom ultra-low attachment plates (Corning Inc., Corning, NY, USA) in organoid growth medium supplemented with 10% Matrigel for 24 hours. Then, the mixtures were embedded in Matrigel and maintained in the organoid complete medium for three days.

**Bulk-RNA sequencing analysis**

Total RNAs from the biopsy sample, PCO, and the mixed PCO-CAF were isolated using Trizol (Thermo Fisher Scientific). After the confirmation of the RNA quality, the construction of the library was performed using QuantSeq 3’ mRNA-Seq Library Prep Kit (Lexogen, Inc., Vienna, Austria) according to the manufacturer’s instructions. Sequencing was performed as single-end sequencing using NextSeq 500 (Illumina, Inc., CA, USA), producing > 10 million reads per sample.

The raw data was removed for adapter sequences using Cutadapt (v1.18)^3^ and were checked using FastQC (v0.11.8). Then, alignment, gene count, and normalization were performed using STAR (v2.7.10a)^4^, FeatureCounts (v2.0.1)^5^, and edgeR (v3.36.0)^6^, respectively. The variable top 500 genes were isolated between each sample, and gene levels were visualized for 4 subtypes according to expression level. The Uniform Manifold Approximation and Projection (UMAP) through these genes were analyzed using umap (v0.2.9.0)^7^ R package, and the ellipses represent using stat ellipse function in ggplot2 (v3.4.0) R package. Gene ontology analysis was performed using clusterProfiler (v4.2.2)^8^ R package. The alignment score and variable top 500 genes were displayed in the (Figure S3 and Table S2).

The RNA-seq data for Pancreatic Adenocarcinoma (PAAD, n=178) dataset were downloaded from The Cancer Genome Atlas (TCGA) by using TCGAbiolinks (v2.29.6).^9^ The counts were normalized by using edgeR (v3.36.0) package, and gene set variants analysis from 4 gene sets was performed by using GSVA (v1.42.0) package.^10^

**Single-cell RNA sequencing**

Single-cell RNA sequencing was performed by using the Chromium Next GEM Single Cell 3' Reagent Kits (10x Genomics Inc., Pleasanton, CA, USA) and was sequenced on a Novaseq 6000 platform according to the manufacturer's instructions. The resulting reads were trimmed and aligned to the GRCh38-2020-A reference genome using Cell Ranger (v7.0) software. Then, data filtering, normalization, and clustering were performed by using the standard analysis pipeline with the R package Seurat (v4.0.3).^11^ Trajectory analysis in the PDAC cluster was conducted by using the standard analysis pipeline of the R package Monocle (v2.22.0).^12^ Significant interactions between cancer cells, myCAFs, and iCAFs were analyzed by using the Cell-Cell database from CellChat (v1.4.0)^13^ R package.

**Drug susceptibility test**

For cell tracing, CAFs were stained with carboxyfluorescein succinimidyl ester (CFSE, Thermo Fisher Scientific) according to the manufacturer’s instructions. The CAFs were mixed with dissociated cancer cells from PCOs. The mixed PCO-CAF culture and the PCO mono-culture were maintained according to the above co-culture methods for 4 days. Subsequently, Gemcitabine (50 nM), Paclitaxel (50 nM), SGX523 (2 μM), Foretinib (2 μM), ANA-12 (10 nM), Sapitinib (5 μM), Trametinib (100 nM), and Binimetinib (1 μM) were treated for 3 days. Following the dissociation of the cells by using TrypLE™ Express (Thermo Fisher Scientific), they were stained with 1 μg/ml propidium iodide (Sigma-Aldrich). FACS analysis was performed to evaluate the viable cells by using Canto II (BD Bioscience, Franklin Lakes, NJ, USA) and FlowJo (v10).

**Flow cytometry analysis**

Dissociated cells were fixed and permeabilized by using the Fixation/Permeabilization wash kit (BioLegend, San Diego, CA, USA). Then, cells were stained with antibodies specific for anti-EpCAM-PE (BioLegend) and anti-Vimentin Alexa Fluor 488 (BioLegend) in the permeabilization buffer. Canto II (BD Bioscience), and FlowJo (v10) were utilized for the analysis.

**Quantitative reverse transcription polymerase chain reaction (qRT-PCR)**

To evaluate effect of epithelial-mesenchymal transition (EMT) by directed co-culture, dissociated cancer cell from organoid were labeled using CellTracker deep red dye (Thermo Fisher Scientific) and paired CAF cells were labeled using CFSE. According to the above co-culture methods, the mixed PCO-CAF and the PCO were cultured for 4 days. The deep red(+)CFSE(-) cancer cells from mixed PCO-CAF were sorted using FACS Aria III (BD Biosciences).

For analysis of paracrine effect through CAF, these were cultured in AdDMEM/F12 medium supplemented with GlutaMAX and 5% FBS for 3 days. The conditioned medium from CAF was collected and filtered through 0.2 µm syringe filter after centrifugation. Organoid for 3 days were incubated using CAF conditioned medium.

The RNA was isolated using the RNeasy Plus Mini Kit (Qiagen, Hilden, Germany), and cDNA was synthesized using GoScript reverse transcription system (Promega, Wisconsin, USA) according to the manufacturer’s instructions. The qRT-PCR was performed using the Thermal Cycler Dice Real Time System III (TaKaRa Bio, Shiga, Japan). The gene level was normalized to a housekeeping gene (*GAPDH*). The primer sequences for Qrt-PCR are as follows:

MMP1-Forward: 5’-TTTGATGCTATAACTACGATTCGGG-3’,

MMP1-Reverse:5’- TGGCCAGAAAACAGAAATGAAATTG-3’,

VIM-Forward:5’- GCAGAAGAATGGTACAAATCCAAG-3’,

VIM-Reverse: 5’-CGGCAAAGTTCTCTTCCATTTCA-3’,

FN1-Forward: 5’-TCACATTTCCAAGTACATTCTCAGG-3’,

FN1-Reverse: 5’-TTTGATGGTGTAGGAGTTTAAGTGG-3’,

ITGA2-Forward: 5’-ACGCTGAAAAATAAAAGGGAAAGTG-3’,

ITGA2-Reverse: 5’-GTAAAAGTCACCTGTTGTTCTCTCT-3’,

LGALS1-Forward: 5’-CAGATGGATACGAATTCAAGTTCCC-3’,

LGALS1-Reverse: 5’-ATTTGATCTTGAAGTCACCGTCAG-3’,

EPCAM-Forward: 5’-AAAGCTGGTGTTATTGCTGTTATTG-3’

EPCAM-Reverse: 5’-CCTTCTCATACTTTGCCATTCTCTT-3’

GAPDH-Forward: 5’-TTGTCAAGCTCATTTCCTGGTATG-3’

GAPDH-Reverse: 5’-TCTCTCTTCCTCTTGTGCTCTTG-3’

**Immunofluorescence staining**

PCOs were stained with CellTracker deep red dye (Thermo Fisher Scientific), and CAFs were stained with CFSE, following the manufacturer's instructions. Deep red-labeled PCOs in the mono-culture and the mixed PCO-CAF system (consisting of deep red-labeled cancer cells with CFSE-labeled CAFs) were cultured for 4 days following the protocol described in “Generation of mixed pancreatic cancer organoid and cancer-associated fibroblast system” section. Then, cells were treated with Gemcitabine (50 nM) and Trametinib (100 nM) and incubated for 3 days. The immunofluorescence staining was performed as previously described, with minor modification.^2^ Briefly, cells were fixed in paraformaldehyde with glutaraldehyde, were washed with phosphate-buffered saline containing 10 mM NaBH_4_ and were stained with cleaved caspase-3 (Asp175) antibody (Cell Signaling Technology, Beverly, MA, USA). The images were obtained by using Nikon AX R confocal microscope (Nikon, Tokyo, Japan) with the 20x CFI Pan Apochromat Lambda objective.

**Statistical analysis**

Data are presented as mean ± SEM. Statistical comparisons between two different groups for drug response were performed using the paired t-test using GraphPad Prism 9 (GraphPad, La Jolla, CA, USA).

**References**

1. Kim S, Woo KJ, Yang CM, et al. Simultaneous establishment of pancreatic cancer organoid and cancer-associated fibroblast using a single-pass endoscopic ultrasound-guided fine needle biopsy specimen. *Dig Endosc*. Jul 31 2023;doi:10.1111/den.14648

2. Choi JI, Jang SI, Hong J, et al. Cancer-initiating cells in human pancreatic cancer organoids are maintained by interactions with endothelial cells. *Cancer Lett*. Feb 1 2021;498:42-53. doi:10.1016/j.canlet.2020.10.012

3. Martin M. Cutadapt removes adapter sequences from high-throughput sequencing reads. *EMBnet journal*. 2011;17(1):10-12.

4. Dobin A, Davis CA, Schlesinger F, et al. STAR: ultrafast universal RNA-seq aligner. *Bioinformatics*. Jan 1 2013;29(1):15-21. doi:10.1093/bioinformatics/bts635

5. Liao Y, Smyth GK, Shi W. featureCounts: an efficient general purpose program for assigning sequence reads to genomic features. *Bioinformatics*. Apr 1 2014;30(7):923-30. doi:10.1093/bioinformatics/btt656

6. Robinson MD, McCarthy DJ, Smyth GK. edgeR: a Bioconductor package for differential expression analysis of digital gene expression data. *Bioinformatics*. Jan 1 2010;26(1):139-40. doi:10.1093/bioinformatics/btp616

7. McInnes L, Healy J, Melville J. Umap: Uniform manifold approximation and projection for dimension reduction. *arXiv preprint arXiv:180203426*. 2018;

8. Wu T, Hu E, Xu S, et al. clusterProfiler 4.0: A universal enrichment tool for interpreting omics data. *Innovation (Camb)*. Aug 28 2021;2(3):100141. doi:10.1016/j.xinn.2021.100141

9. Colaprico A, Silva TC, Olsen C, et al. TCGAbiolinks: an R/Bioconductor package for integrative analysis of TCGA data. *Nucleic Acids Res*. May 5 2016;44(8):e71. doi:10.1093/nar/gkv1507

10. Hanzelmann S, Castelo R, Guinney J. GSVA: gene set variation analysis for microarray and RNA-seq data. *BMC Bioinformatics*. Jan 16 2013;14:7. doi:10.1186/1471-2105-14-7

11. Hao Y, Hao S, Andersen-Nissen E, et al. Integrated analysis of multimodal single-cell data. *Cell*. Jun 24 2021;184(13):3573-3587 e29. doi:10.1016/j.cell.2021.04.048

12. Trapnell C, Cacchiarelli D, Grimsby J, et al. The dynamics and regulators of cell fate decisions are revealed by pseudotemporal ordering of single cells. *Nat Biotechnol*. Apr 2014;32(4):381-386. doi:10.1038/nbt.2859

13. Jin S, Guerrero-Juarez CF, Zhang L, et al. Inference and analysis of cell-cell communication using CellChat. *Nat Commun*. Feb 17 2021;12(1):1088. doi:10.1038/s41467-021-21246-9
