# Supplementary material for: Bidirectional crosstalk between cancer cells and cancer‐associated fibroblasts in mixed organoid system elicits transcriptomic characteristics of pancreatic cancer with potential therapeutic vulnerabilities
Source: Clin Transl Med. 2024 Feb 22;14(2):e1597. doi: 10.1002/ctm2.1597 (PMC10883236; doi:10.1002/ctm2.1597)
Supplement: Supplementary file 2 — Supporting Information [file CTM2-14-e1597-s002.docx]

**Supplementary Table**

**Table S1** Clinicopathologic characteristics of the patients.

| Sample | Sex | Age | Histologic diagnosis | Tumor size | Tumor location | Clinical stage | Clinical TNM stage | Mutation status |
| --- | --- | --- | --- | --- | --- | --- | --- | --- |
| 52 | F | 73 | ADC, P/D | 4.0cm | Body | IV | T4N1M1 | KRAS G12D |
| 53 | F | 72 | ADC, W/D | 3.7cm | Tail | IV | T2N1M1 | KRAS G12D |
| 55 | F | 72 | ADC, M/D | 4.0cm | Head | IIA | T3N0M0 | KRAS G12R |
| 70 | M | 70 | ADC, M/D | 5.0cm | Tail | IV | T3N2M1 | KRAS G12D |
| 79 | M | 64 | ADC, P/D | 4.4cm | Body | IV | T4N2M1 | KRAS G12D |

W/D, well differentiated; M/D, moderately differentiated, P/D, poorly differentiated.

**Table S2** The top 500 variable genes for patient tissue, mixed pancreatic cancer organoid (PCO)-cancer-associated fibroblast and PCO.

| **Gene set 1** | **Gene set 2** | **Gene set 3** | **Gene set 4** |
| --- | --- | --- | --- |
| HBB | RPS4Y1 | COL1A2 | XIST |
| HBA2 | DDX3Y | IGFBP5 | CPB1 |
| HBA1 | HLA-DRB5 | COL3A1 | REG1A |
| S100A8 | USP9Y | TFPI2 | PGC |
| S100A9 | ANKRD1 | FN1 | CPA1 |
| IGKC | KRT13 | COL6A3 | CLPS |
| TYROBP | KLK6 | DCN | CTRB2 |
| SPP1 | SLC28A2 | SPARC | REG3A |
| PTPRC | KLK7 | IGFBP7 | PLA2G1B |
| FYB1 | CDKN2A | INHBA | PRSS2 |
| LAPTM5 | SULT1C2 | COL1A1 | OLFM4 |
| MNDA | TXLNGY | COL6A2 | PNLIP |
| LCP1 | ALDH3A1 | CHI3L1 | CTRB1 |
| SPARCL1 | HPN | NEFM | PRSS1 |
| CYBB | EIF1AY | THBS2 | SPINK1 |
| KRT17 |  | POSTN | GP2 |
| ITGB2 |  | SERPINE1 | CTRC |
| C3 |  | PAPPA | REG1B |
| CD37 |  | VCAN | CEL |
| FCGR3B |  | BGN | BPIFB1 |
| EVI2B |  | MEG3 | PNLIPRP2 |
| CSF3R |  | MAP1B | CPA2 |
| ONECUT2 |  | STC1 | MSMB |
| SELL |  | SRGN | CELA2B |
| IGHM |  | PTGS2 | CELA2A |
| COL11A1 |  | COL6A1 | SYCN |
| GIMAP4 |  | PDGFRB | INS |
| C1QA |  | MEDAG | PNLIPRP1 |
| AIF1 |  | COL4A1 | TTN |
| FOSB |  | COL5A1 | VSIG1 |
| PRKCB |  | FBN1 | CD70 |
| BCL2A1 |  | APOD | CFTR |
| FCN1 |  | SFRP1 | IGLC3 |
| CXCL14 |  | C11orf96 | LOC100506281 |
| IGHG4 |  | PDGFRA | IGHG3 |
| RGS2 |  | ZEB2 | REG3G |
| PLXDC2 |  | MT2A | GCG |
| SFRP2 |  | SERPINE2 | NTRK2 |
| LY6D |  | DDR2 |  |
| ARHGAP30 |  | COL4A2 |  |
| RIPOR2 |  | PXDN |  |
| CXCR2 |  | PRG4 |  |
| PPBP |  | TIMP3 |  |
| C1QB |  | LOXL2 |  |
| FCGR3A |  | SGIP1 |  |
| IGSF6 |  | WNT5A |  |
| NCKAP1L |  | PLAT |  |
| A2M |  | FAP |  |
| SFRP4 |  | BASP1 |  |
| FCER1G |  | COL5A2 |  |
| ALOX5AP |  | MAP1A |  |
| GIMAP7 |  | GPNMB |  |
| COL10A1 |  | MGP |  |
| FGL2 |  | MIR100HG |  |
| MS4A1 |  | LUM |  |
| FCGR2A |  | MMP2 |  |
| PIGR |  | ITGA11 |  |
| PECAM1 |  | MYLK |  |
| CD52 |  | PRRX1 |  |
| FPR1 |  | VIM |  |
| IGHA1 |  | GYPC |  |
| PADI1 |  | GREM1 |  |
| PF4 |  | IGFBP3 |  |
| CD14 |  | COL8A1 |  |
| APBB1IP |  | CCDC80 |  |
| WAS |  | CLMP |  |
| MYO1F |  | SULF1 |  |
| CCL5 |  | SCG2 |  |
| BIN2 |  | MRC2 |  |
| CD93 |  | MUCL3 |  |
| HLA-DPA1 |  | CELF2 |  |
| NCF2 |  | FLRT2 |  |
| PLEK |  | MAFB |  |
| VNN2 |  | SLC2A3 |  |
| HCLS1 |  | MXRA8 |  |
| CD53 |  | LTBP2 |  |
| SST |  | MMP1 |  |
| CD84 |  | UCHL1 |  |
| IL7R |  | IL33 |  |
| MUC6 |  | CPE |  |
| IGLC2 |  | DUOX2 |  |
| HEMGN |  | IRAG1 |  |
| MS4A7 |  | DIO2 |  |
| CLEC2B |  | THY1 |  |
| SPN |  | ZEB1 |  |
| ITGAX |  | EDIL3 |  |
| LST1 |  | CLDN11 |  |
| IKZF1 |  | EMILIN1 |  |
| SLC11A1 |  | COL5A3 |  |
| JCHAIN |  | AEBP1 |  |
| HLA-DQB1 |  | LAMA4 |  |
| IL10RA |  | AQP1 |  |
| MT1G |  | CST2 |  |
| ARHGAP25 |  | SERPING1 |  |
| TMEM176B |  | TAGLN |  |
| LILRB3 |  | BDKRB2 |  |
| TRAC |  | CDH11 |  |
| GMFG |  | C7 |  |
| JAML |  | CSF3 |  |
| CCR1 |  | SCUBE3 |  |
| APOC1 |  | CDH2 |  |
| LILRB2 |  | FKBP10 |  |
| PLCB2 |  | BNC2 |  |
| CXCL17 |  | CXCL6 |  |
| MS4A6A |  | COL15A1 |  |
| SLA |  | HSPB6 |  |
| KRT6A |  | VCAM1 |  |
| MARCHF1 |  | TWIST1 |  |
| S100A2 |  | SNED1 |  |
| SPOCK2 |  | ABI3BP |  |
| DEFB1 |  | PLA2G2A |  |
| RGS1 |  | PDE3A |  |
| CP |  | FGF7 |  |
| REG4 |  | SPON2 |  |
| LSP1 |  | FSTL1 |  |
| HLA-DPB1 |  | PCOLCE |  |
| HLA-DQA1 |  | WIPF1 |  |
| ARHGAP9 |  | SYNE1 |  |
| IFITM2 |  | C1R |  |
| TRAF3IP3 |  | PLIN2 |  |
| MRC1 |  | MAF |  |
| TREM1 |  | GJA1 |  |
| ASPN |  | IL1B |  |
| RASSF2 |  | LRRC32 |  |
| ALAS2 |  | RGS4 |  |
| CLEC4E |  | DSEL |  |
| MUC5B |  | BDKRB1 |  |
| LCP2 |  | CDH13 |  |
| GBP5 |  | PLXDC1 |  |
| TSC22D3 |  | PTGES |  |
| CCL3 |  | TGFBI |  |
| SPON1 |  | PTGDS |  |
| CLEC7A |  | EDNRA |  |
| FGR |  | AKT3 |  |
| SP140 |  | IL11 |  |
| TUBB1 |  | SSC5D |  |
| S100A12 |  | SEMA5A |  |
| LAIR1 |  | MYL9 |  |
| ITGAL |  | AREG |  |
| IFI44L |  | ITGA1 |  |
| FAM83A |  | COL14A1 |  |
| FLT1 |  | TCF4 |  |
| TMEM176A |  | CCL2 |  |
| DUSP1 |  | SERPINF1 |  |
| LILRB1 |  | NTM |  |
| CD163 |  | ENG |  |
| RUNX3 |  | NOVA1 |  |
| TM4SF4 |  | APOE |  |
| SLPI |  | NR2F1 |  |
| CYTH4 |  | LOX |  |
| TRBC2 |  | G0S2 |  |
| WFDC2 |  | SUSD2 |  |
| GPR65 |  | LMOD1 |  |
| VSIG4 |  | CEACAM5 |  |
| DOCK8 |  | IL6 |  |
| MUC4 |  | ANGPTL4 |  |
| SPINK4 |  | TBX2 |  |
| HCK |  | GNG11 |  |
| IL16 |  | EBF1 |  |
| CAVIN2 |  | ADAMTS12 |  |
| GBP4 |  | AKR1C1 |  |
| INPP5D |  | PDZRN3 |  |
| CD2 |  | TMEM158 |  |
| NCF1C |  | TMEM132B |  |
| EVI2A |  | BCAT1 |  |
| CFI |  | NID1 |  |
| MSR1 |  | LRRK2 |  |
| LEFTY1 |  | PLCB1 |  |
| TNFSF14 |  | SNORD13 |  |
| KCNJ15 |  | LUCAT1 |  |
| DDX43 |  | ARHGAP22 |  |
| ARHGDIB |  | TBX3 |  |
| HLA-DRA |  | SPOCK1 |  |
| ITLN1 |  | EREG |  |
| TAGAP |  | NPTX1 |  |
| PRSS3 |  | CLEC11A |  |
| PSCA |  | CXCL12 |  |
| ADH1C |  | ADAMTS5 |  |
| CD86 |  | RFTN1 |  |
| NCF4 |  | POU2F2 |  |
| SERPINB5 |  | ISLR |  |
| SERPINB3 |  | C1S |  |
| FMNL1 |  | SERPINB2 |  |
| SOX2 |  | MEF2C |  |
| P2RY13 |  | MMP11 |  |
| F13A1 |  | FBLN1 |  |
| MYH11 |  | GFPT2 |  |
|  |  | FAM20A |  |
|  |  | PALM2AKAP2 |  |
|  |  | SLC22A17 |  |
|  |  | LGALS1 |  |
|  |  | ITGA7 |  |
|  |  | C14orf132 |  |
|  |  | ZFHX4 |  |
|  |  | RUNX1T1 |  |
|  |  | ADAMTS4 |  |
|  |  | RASD1 |  |
|  |  | ZMAT1 |  |
|  |  | SCN9A |  |
|  |  | NNMT |  |
|  |  | TNC |  |
|  |  | TNS1 |  |
|  |  | SDC2 |  |
|  |  | TMEM47 |  |
|  |  | PIEZO2 |  |
|  |  | ADM |  |
|  |  | ABCA9 |  |
|  |  | PTX3 |  |
|  |  | HAND2-AS1 |  |
|  |  | COL12A1 |  |
|  |  | ABCA8 |  |
|  |  | RFLNB |  |
|  |  | KCNMA1 |  |
|  |  | SIRPA |  |
|  |  | MFAP5 |  |
|  |  | DYSF |  |
|  |  | TCIM |  |
|  |  | DCBLD2 |  |
|  |  | ANPEP |  |
|  |  | HAS1 |  |
|  |  | ELN |  |
|  |  | PAX8-AS1 |  |
|  |  | CAV1 |  |
|  |  | PTGIS |  |
|  |  | EFEMP1 |  |
|  |  | CHST2 |  |
|  |  | ACTA2 |  |
|  |  | PREX1 |  |
|  |  | CACNA1A |  |
|  |  | SLC14A1 |  |
|  |  | ITGBL1 |  |
|  |  | CD248 |  |
|  |  | COL7A1 |  |
|  |  | DZIP1 |  |
|  |  | NDN |  |
|  |  | RAB34 |  |
|  |  | COX7A1 |  |
|  |  | CFH |  |
|  |  | PLXNC1 |  |
|  |  | NRG1 |  |
|  |  | ITGB3 |  |
|  |  | BICC1 |  |
|  |  | SRPX |  |
|  |  | SPOCD1 |  |
|  |  | SETBP1 |  |
|  |  | PLAC9 |  |
|  |  | HGF |  |
|  |  | MMP3 |  |
|  |  | SLIT2 |  |
|  |  | TIMP1 |  |
|  |  | PAMR1 |  |
|  |  | MPDZ |  |
|  |  | PTGIR |  |
|  |  | HEG1 |  |
|  |  | CST4 |  |
|  |  | SLC6A15 |  |
|  |  | FAM126A |  |
|  |  | KLF7 |  |
|  |  | SLITRK4 |  |
|  |  | ABCA6 |  |
