# Supplementary material for: Bidirectional crosstalk between cancer cells and cancer‐associated fibroblasts in mixed organoid system elicits transcriptomic characteristics of pancreatic cancer with potential therapeutic vulnerabilities
Source: Clin Transl Med. 2024 Feb 22;14(2):e1597. doi: 10.1002/ctm2.1597 (PMC10883236; doi:10.1002/ctm2.1597)
Supplement: Supplementary file 3 — FiguresS1‐S5 [file CTM2-14-e1597-s001.docx]

**Supplementary Figures**

**
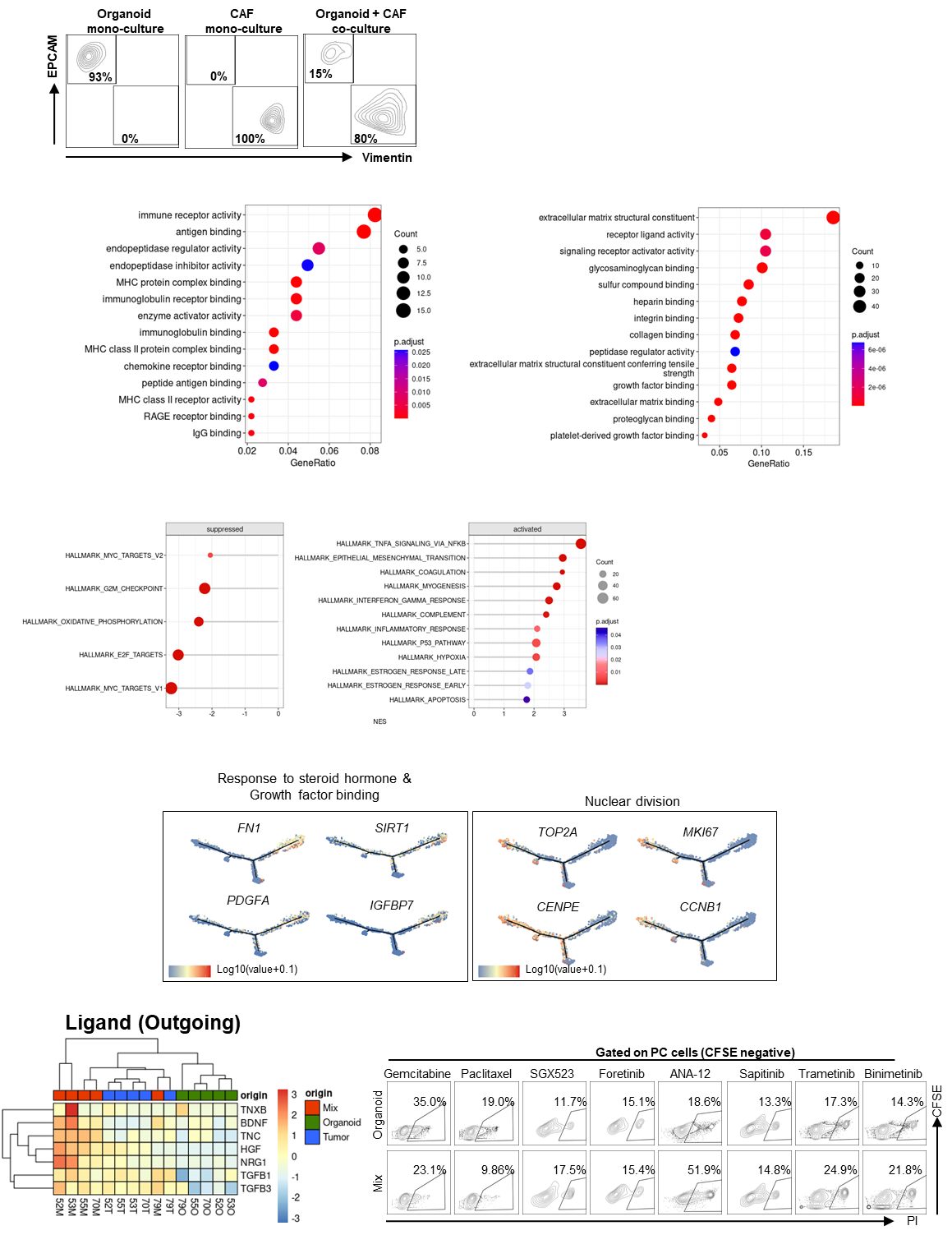
**

**Figure S1** Flow cytometric analysis of the pancreatic cancer organoid (PCO) mono-culture, the cancer-associated fibroblast (CAF) mono-culture, and the PCO-CAF co-culture for evaluating the epithelial cell component (EpCAM) and the mesenchymal cell component (vimentin).


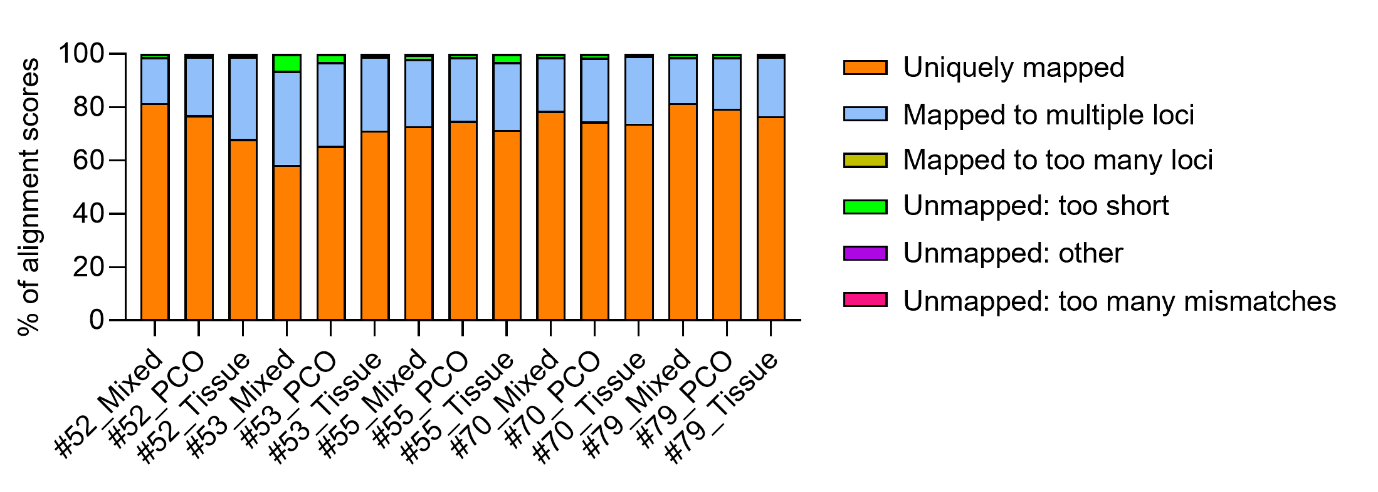


**Figure S2** Alignment efficiency for bulk RNA-sequencing for patient tissue, mixed pancreatic cancer organoid (PCO)-cancer-associated fibroblasts, and PCO mono-cultures.

**
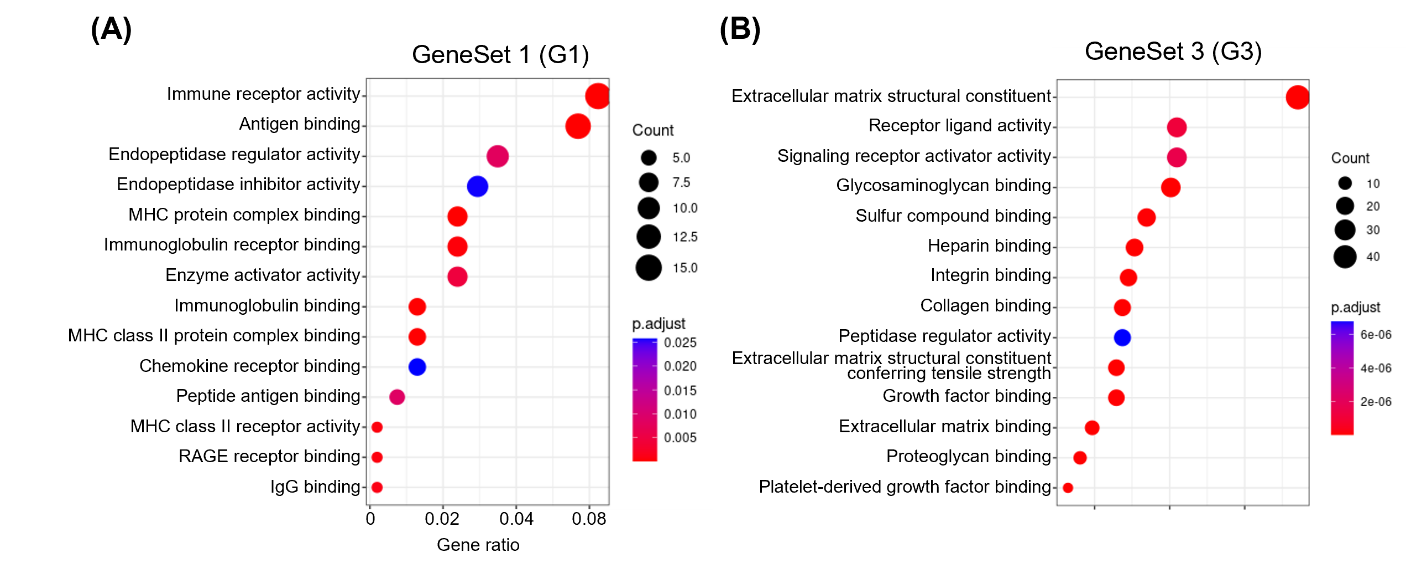
**

**Figure S3** (A) Enriched gene ontology in Geneset 1 including 187 genes. (B) Enriched gene ontology in Geneset 3 including 260 genes.


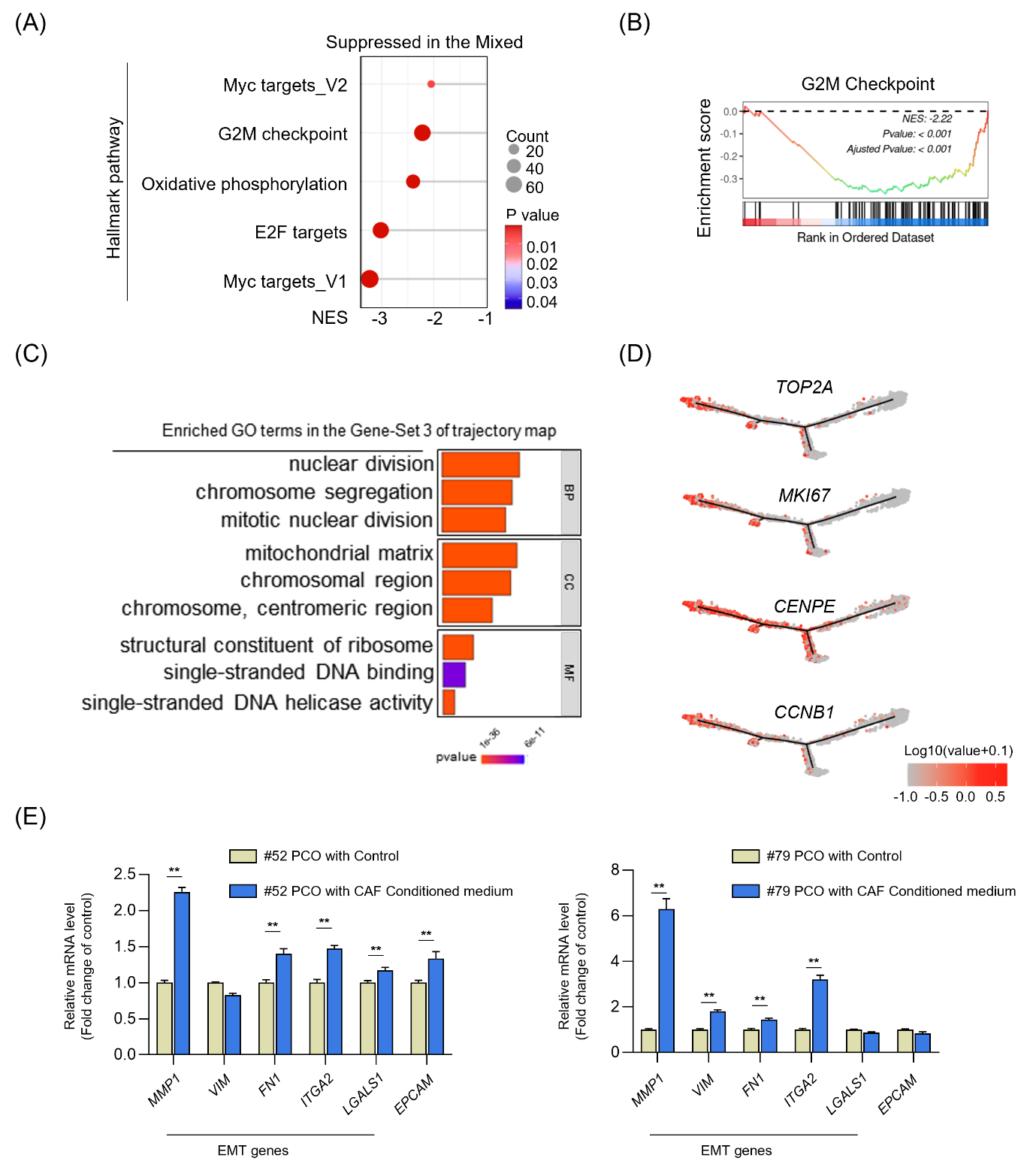


**Figure S4** (A) Suppressed Hallmark gene sets in pancreatic cancer cells in the mixed pancreatic cancer organoid (PCO) and cancer-associated fibroblast culture compared to the PCO mono-culture by gene set enrichment analysis. (B) Gene Set Enrichment Analysis (GSEA) of G2M Checkpoint gene set in the mixed pancreatic cancer organoid (PCO) and cancer-associated fibroblast (CAF) culture compared to the PCO mono-culture. The results revealed enrichment of G2M Checkpoint gene set in the PCO mono-culture. (C) Enriched gene ontology in the cancer cells of pancreatic cancer-only organoid in trajectory analysis. (D) Trajectory maps of the representative genes in the ontology of " Nuclear division” (GO:0000280)”, enriched in the pancreatic cancer-only organoid. (E) The gene levels in the #79 and #52 organoids treated for 3 days with CAF conditioned medium.

**
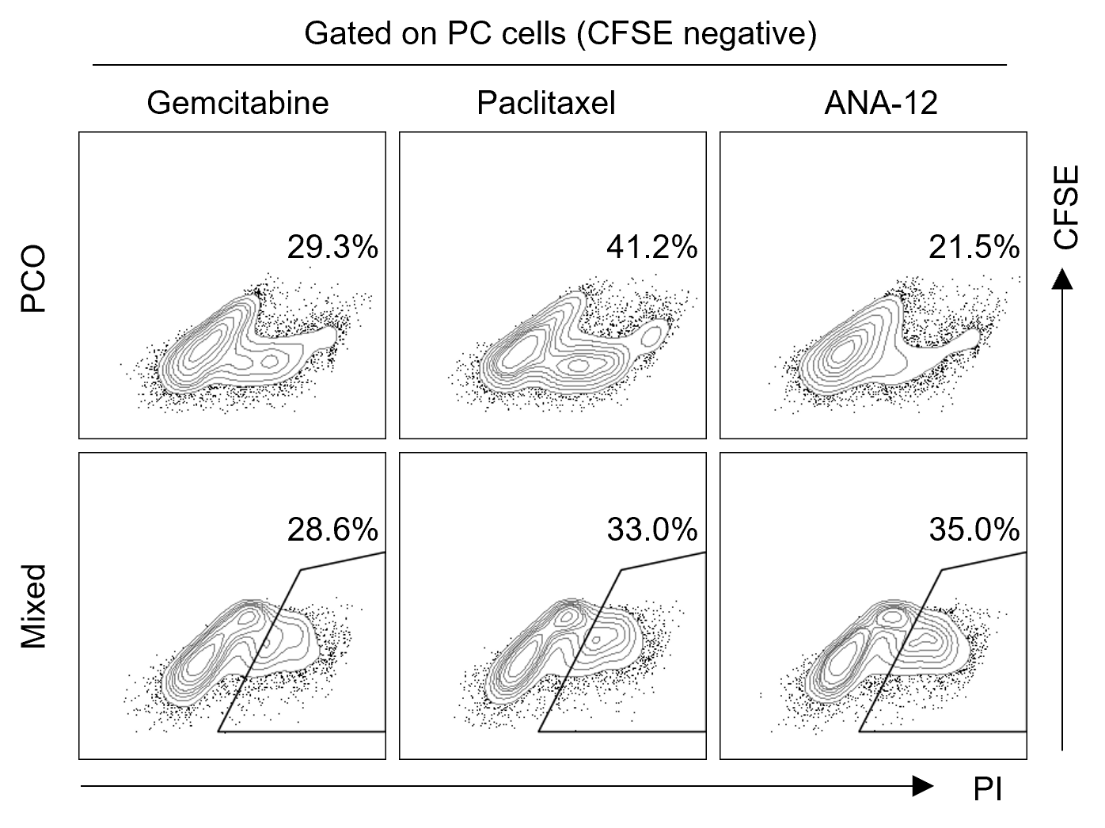
**

**Figure S5** FACS plot and the relative drug response in the mixed pancreatic cancer organoid (PCO) and cancer-associated fibroblast culture and the PCO mono-culture from the patient #70.
